# Differences in Pathophysiology and Treatment Efficacy Based on Heterogeneous Out-of-Hospital Cardiac Arrest

**DOI:** 10.3390/medicina60030510

**Published:** 2024-03-21

**Authors:** Shu Utsumi, Mitsuaki Nishikimi, Shinichiro Ohshimo, Nobuaki Shime

**Affiliations:** Department of Emergency and Critical Care Medicine, Graduate School of Biomedical and Health Sciences, Hiroshima University, Hiroshima 734-8551, Japan; asap903@hiroshima-u.ac.jp (S.U.); ohshimos@hiroshima-u.ac.jp (S.O.)

**Keywords:** cardiopulmonary arrest, diversity, therapeutic hypothermia

## Abstract

Out-of-hospital cardiac arrest (OHCA) is heterogeneous in terms of etiology and severity. Owing to this heterogeneity, differences in outcome and treatment efficacy have been reported from case to case; however, few reviews have focused on the heterogeneity of OHCA. We conducted a literature review to identify differences in the prognosis and treatment efficacy in terms of CA-related waveforms (shockable or non-shockable), age (adult or pediatric), and post-CA syndrome severity and to determine the preferred treatment for patients with OHCA to improve outcomes.

## 1. Introduction

Out-of-hospital cardiac arrest (OHCA) is a leading cause of mortality, affecting over 350,000 individuals in the United States every year [1,2,3,4]. Despite developments in resuscitation science, including those within the educational system [5,6,7] and in pre-hospital management [8,9], survival rates remain low. The 2021 European Resuscitation Council Guidelines state the following: “Survival rates at hospital discharge are on average 8%, varying from 0% to 18%” (Page 62 Paragraph 5 Line 13) [1]. Even when patients do survive, poor functional outcomes often affect their reintegration into society. European studies have reported that the proportion of patients with poor neurological outcomes was >50% in situations where withdrawal of life sustaining treatment (WLST) was not applied; however, it was <10% in countries where WLST was routinely performed [1]. A recent United States study reported that approximately 20% of survivors have poor neurological outcomes [2]. Thus, OHCA remains an important issue not only in terms of its high mortality rate but also in relation to the neurological prognosis of survivors.

Various therapeutic interventions have been investigated for patients with OHCA to improve prognosis, such as medication during resuscitation, airway management, and intensive care, with a focus on temperature management after the return of spontaneous circulation (ROSC) [1,2,3,4,5,6,7,8,9]. OHCA is heterogeneous in many ways, including the cause of cardiac arrest (CA), age, and the severity of hypoxic encephalopathy, and treatment effects and prognosis vary from case to case [10,11,12]. Therefore, it is important to select the appropriate therapeutic intervention for each patient with CA. We conducted a review on the heterogeneity of OHCA in terms of CA waveform (shockable or non-shockable), age (adult or child), and the differential effects of post-resuscitation therapies. Our study focused on the major heterogeneous elements of these patients with CA, with the aim of detecting effective therapeutic interventions and improving their prognosis.

### 1.1. Shockable vs. Non-Shockable Rhythms: Pathophysiology, Prognosis, and Treatment Response

In 1997, Vaagenes et al. first described differences in morphological patterns of brain damage between shockable ventricular fibrillatory CA (VFCA) and non-shockable asphyxial CA (ACA) models [13]. In their study, VFCA models showed scattered ischemic neuronal changes in multiple brain regions, while ACA models showed both scattered ischemic neuronal changes and microinfarcts. This initial investigation led to further animal studies on pathophysiological differences linked to CA waveforms [14] (Table 1).

We reviewed animal studies published in the last decade that compared ACA and VFCA. In 2013, Li et al. investigated the difference between ACA and VFCA in terms of brain damage after CA [15]. Healthy male rats were randomly assigned to ACA (n = 15), VFCA (n = 15), or sham (n = 5) groups, and all groups underwent CPR after 6 min of CA. Brain damage was assessed with respect to the neurological deficit score (NDS), a tape removal test (TRT) to assess sensory–motor integration, and serum S-100B concentration on days 1, 3, and 7 after ROSC. In addition, brain specimens were retrieved and evaluated using a histopathological damage scoring system. Serum S-100B levels at 1, 3, and 7 days were significantly higher in the ACA group than in the VFCA group; however, no differences in NDS or TRT were observed between the two groups. The histopathological damage scores were also significantly higher in the ACA group. Thus, Li et al. concluded that post-CA brain damage differed between the two groups because of differences in serum S-100B and morphological brain damage. Specifically, they concluded that the NDS and TRT can be used as tools to assess brain damage from a functional perspective; there were differences in brain damage in functional areas only, which may have had an effect; there were differences in brain damage after CA between the ACA and VFCA groups; and there were differences in the interventions involved in brain resuscitation. They concluded that there were differences in brain injury after ACA and VFCA and differences in the interventions involved in brain resuscitation. Wu et al. compared myocardial dysfunction after ROSC between ACA and VFCA in a porcine CA model [16]. They randomly divided 32 pigs into a VFCA (n = 16) or an ACA (n = 16) group and performed cardiopulmonary resuscitation (CPR) following 8 min of CA, induced using electrical stimulation or endotracheal tube clamping. At 1, 2, 4, and 6 h post-ROSC, myocardial injury was assessed using echocardiography, myocardial perfusion imaging, and transmission electron microscopy. The ACA group showed worse cardiac dysfunction and myocardial injury, a lower rate of ROSC (ACA, 50% vs. VFCA, 100%), and shorter survival time compared with the VFCA group (ACA, 2.4 ± 0.9 h vs. VFCA, 5.7 ± 0.2 h). They proposed that this may have been because of hypoxia and acidosis during asphyxia, which can cause severe metabolic disturbances. However, Uray et al. reported contrasting results among rat models [17], which may be attributed to differences in experimental methods and CA duration. In their study, 25 rats were randomly classified as VFCA (n = 10), ACA (n = 10), and sham (n = 5), and CPR was performed after 5 min of CA. Immediately post-ROSC, cardiac function evaluation was continued using ultrasonography for 30 min to assess differences between the VFCA and ACA groups. The results showed that the cardiac output was higher in the ACA group than in the VFCA group. The study also compared neurological deficits between the VFCA and ACA groups using the neurological deficit score on days 1, 2, 3, and 8 post-ROSC. The ACA group reported significantly worse neurological deficits than the VFCA group.

**Table 1:** Laboratory studies evaluating pathophysiological differences between ACA and VFCA after resuscitation.

Study	Year	Animal Model	Duration of CA and CPR	Evaluation Item and Timing	Results
Lin et al. [15]	2013	Dog	CA: 6 min. CPR: Until ROSC	Brain damage At after 1, 3, 7 days after ROSC Histopathologic evaluation	ACA exacerbated morphological brain damage compared to VFCA.
Wu et al. [16]	2013	Swine	CA: 8 min. CPR: Until ROSC or 30 min	Cardiac dysfunction At 1, 2, 4, 6 h after ROSC	Myocardial dysfunction after ACA is more severe than with VFCA.
Drabek et al. [18]	2014	Rat	CA: 8 min. CPR: 5 min	Cerebral blood flow For 1 h immediately after ROSC	ACA showed early perfusion enhancement in the cortex and thalamus after ROSC, while VFCA showed early perfusion enhancement only in the cortex.
Varvarousis et al. [19]	2016	Swine	CA: 5 min. CPR: Until ROSC or 30 min	Metabolic profiling During CA and CPR At 1, 2, 3, 4, 24 h after ROSC	ACA showed significant metabolic disturbances during the asphyxial and CA phases, while for VFCA animals at the resuscitation phase.
Uray et al. [17]	2018	Rat	CA: 5 min. CPR: Until ROSC	Cardiac dysfunction Not stated Neurologic injury At 1, 3, 5, 8 days after ROSC	Cardiac dysfunction was significantly more severe in the VFCA group and neurological injury was significantly worse in the ACA group.

ACA, asphyxial cardiac arrest; VFCA, ventricular fibrillation cardiac arrest; CA, cardiac arrest; CPR, cardiac pulmonary resustation.

In 2014, Drabek et al. compared post-ROSC cerebral blood flow (CBF) reperfusion patterns between ACA and VFCA [18]. Adult male rats were randomly classified as VFCA (n = 23) or ACA (n = 21) and were untreated for 8 min after CA, followed by CPR for a maximum resuscitation time of 5 min until ROSC. CBF was assessed in four regions, namely, the cortex, thalamus, hippocampus, and amygdala/pisiform complex, for 60 min immediately post-ROSC. They found that the ACA group showed early hyperperfusion in the cortex and thalamus, whereas the VFCA group showed early hyperperfusion in the cortex only. The underlying molecular mechanism remains unknown; however, the authors suggested that differences in CBF reperfusion patterns may, at least partially, be explained by CA onset. The VFCA model involves a sudden CA onset (complete cessation of blood flow followed by electrical stimulation), which contrasts with the gradual CA onset in ACA. Moreover, they suggested that these results may indicate different therapeutic target regions (or mechanisms) and mechanisms of post-ROSC brain injury between the two models, warranting further investigations. In 2016, Varvarousis et al. identified contrasting metabolomic profiles in post-CA and post-ROSC plasma samples between ACA and VFCA [19], which supports the possibility of different mechanisms for post-CA brain injury. That study showed that arginine levels decreased during resuscitation in ACA, contrary to the stable levels observed in VFCA. This finding suggests the potential role of nitric oxide (NO) production and vasodilation in mitigating ACA-related brain injury, considering that arginine is crucial in NO production. Furthermore, NO generation has been reported to be generally associated with post-CA brain injury in previous studies [20,21]. Therefore, the NO metabolic pathway shows potential as a therapeutic target after CA.

Several studies have consistently shown better prognoses (survival and neurological outcomes) for shockable CA compared with non-shockable CA [1,2,3,4]. The most recent study supporting this finding was undertaken by Havranek et al. in 2022 [22]. Interestingly, differences in the validity and thresholds of prognostic indicators have been reported between shockable and non-shockable initial waveforms. Lah et al. compared exhaled end-expiratory partial pressure of carbon dioxide (PetCO_2_) between patients with ACA in non-shockable rhythm and patients with primary CA (acute myocardial infarction or malignant arrhythmias) in shockable rhythm [23]. This prospective observational study included 51 patients with ACA and 63 patients with primary CA (VF or ventricular tachycardia) at two emergency medical centers in Slovenia and Maribor. PetCO_2_ was measured every minute immediately after tracheal intubation during resuscitation and continued until ROSC or CPR interruption. The initial PetCO_2_ values were reported to be significantly higher in the asphyxial CA group (ACA 6.74 ± 4.22 kilopascals (kPa) vs. primary CA 5.1 ± 2.47 kPa), and the trend continued up to 3 min after initiation of CPR. However, after 3 min, no differences between patients with ACA and primary CA were observed. They explained their findings in terms of the higher initial PetCO_2_ in patients with ACA being due to CA via ventilatory failure and that resuscitation would have eliminated the difference. Thus, their findings suggest different PetCO_2_ kinetics between patients with ACA with non-shockable rhythms and patients with primary CA with shockable rhythms. The difference in PetCO_2_ values between patients with ROSC and those without ROSC was also evaluated in each group. In the ACA group, no significant difference in initial PetCO_2_ values was observed between the ROSC and no ROSC groups (ROSC, 6.96 ± 3.63 kPa vs. no ROSC, 5.77 ± 4.64 kPa), whereas in the primary CA group, PetCO_2_ was found to be significantly higher in patients who achieved ROSC (ROSC, 4. 62 ± 2.46 kPa vs. no ROSC, 3.29 ± 1.76 kPa). Based on these findings, the initial PetCO_2_ value may be a useful prognostic indicator for patients with primary CA but not for those with ACA. In the clinical setting, it may be necessary to consider that patients with ACA may show favorable outcomes even if they have higher initial PetCO_2_ values; however, further studies are needed.

In 2023, Kim et al. compared the predictive accuracies of serum neuron-specific enolase (NSE) for neurological outcomes between shockable and non-shockable CA [24]. This study compared NSE using the initial waveform (shockable or non-shockable) in a registry of adult OHCA survivors treated with targeted temperature management (TTM) at 22 academic hospitals in Korea. NSE was measured at 48 h after ROSC. In total, 623 patients were included; 245 had initial shockable CAs and 378 had non-shockable CAs. The median NSE values were significantly higher in the non-shockable group than in the shockable group (104.6 [40.6–228.4] vs. 25.9 [16.7–53.4] ng/mL, respectively). Furthermore, the predictive ability of NSE for poor prognosis, as assessed using the area under the receiver operating characteristic curve, was significantly higher in the non-shockable group than in the shockable group (0.92 vs. 0.86, respectively). The NSE cut-off value for a false positive rate < 1% was also different for the two groups (69.3 [sensitivity 42.1%] vs. 102.7 [sensitivity 76%] ng/mL, respectively). The following factors were discussed as possible reasons for the difference in the prognostic ability of NSE and its cut-off value depending on the initial waveform. First, there was a difference in cause-of-death between patients with OHCA with an initial shockable rhythm (circulatory failure) and those with a non-shockable rhythm (neurologic injury). Second, the prognostic value of NSE is dependent on resuscitation time, which tends to be longer in ACA than in VFCA. Lastly, VFCA carries a higher risk of post-awakening mortality due to persistent circulatory failure or cardiac ischemia, which reduces the predictive ability of NSE.

Regarding treatment during resuscitation, the association between the initial waveform and treatment response has been evaluated. Several high-quality studies on adrenaline and prehospital advanced airway management (AAM) have recently been reported. A meta-analysis of two high-quality randomized controlled trials (RCTs) evaluated the effects of adrenaline according to differences in the initial waveforms [25]. The RCTs were based on the PARAMEDIC-2 trial, which was a multicenter, double-blind, placebo-controlled trial conducted by five National Health Service ambulance services in the United Kingdom from December 2014 to October 2017, and the PACA trial, a double-blind, randomized, placebo-controlled trial conducted in Australia from August 2006 to November 2009, involving patients with OHCA. The meta-analysis evaluated the effect of adrenaline vs. a placebo for each initial waveform, with survival at discharge and neurological prognosis as the outcomes. The pooled odds of survival in patients with non-shockable CA increased with adrenaline use (adjusted odds ratio [aOR] 2.57, 95% confidence interval [CI] 1.36–4.83), whereas it did not differ from placebo in patients with shockable CA (aOR 1.26, 95% CI 0.93–1.71). There was no difference in the neurological prognosis between adrenaline and placebo regardless of the initial waveform.

A network meta-analysis conducted in 2023 evaluated the effects of adrenaline, including subgroup analysis of the initial waveform [26]. This network meta-analysis included 21,594 patients from 18 RCTs and assessed survival and neurological outcomes at discharge. It found that standard-dose epinephrine improved survival to hospital discharge in non-shockable CA compared with placebo or no treatment (aOR 2.10, 95% CI 1.21–3.63) but not in shockable CA (aOR 0.85, 95% CI 0.39–1.85), similar to a previous report [24]. Moreover, the neurological outcome showed no improvement with adrenaline, consistent with that previous report [24].

These differential effects of epinephrine, depending on the initial waveform, might be explained by differences in the underlying mechanisms. Given the background of cardiac disease in patients with shockable CA, epinephrine has been associated with increased myocardial oxygen demand, increased incidence of recurrent CA, and worse myocardial dysfunction after ROSC, suggesting that patients with shockable CA may benefit less from epinephrine than those without non-shockable CA [27].

Regarding AAM, Izawa et al. reported that the effect of each initial waveform was different in 2020 in Japan. Using data from the All-Japan Utstein Registry database, this large cohort study comprised 310,620 adult patients with OHCA. The main outcome was survival at 1 month or within 1 month of discharge. They reported that prehospital AAM improved mortality rates in non-shockable CA (adjusted risk ratio [aRR] 0.87, 95% CI 0.79–0.96) but not in shockable CA (aRR 1.00, 95% CI 0.93–1.07) [28]. This study also evaluated favorable neurological outcomes (defined as a cerebral performance category scale of 1 or 2) at 1 month or at hospital discharge within 1 month. Neither supraglottic airway [SGA] (aRR 0.89, 95% CI 0.81–0.99) nor endotracheal [ET] intubation (aRR 0.75, 95% CI 0.56–1.00) improved the neurological outcome in shockable CA, whereas ET intubation improved the neurological outcome in non-shockable CA (aRR 1.46, 95% CI 1.09–1.96). In 2021, Okubo et al. analyzed data from the All-Japan Utstein database to investigate the effect of prehospital AAM in 424,260 patients per initial waveform, considering the timing of implementation (30 min from EMS contact, divided into 5-min intervals) [29]. For shockable CA, the RRs (95% CI) of AAM to 1-month survival were 1.01 (0.89–1.15) at 0–5 min, 1.06 (0.98–1.15) at 5–10 min, 0.99 (0.87–1.12) at 10–15 min, 0.74 (0.59–0.92) at 15–20 min, 20–25 min, 0.61 (0.37–1.00), and 0.73 (0.26–2.07) for 25–30 min, indicating a negative effect of prehospital AAM. In contrast, for non-shockable CA, the RRs for AAM were 1.12 (1.00–1.27) for 0–5 min, 1.34 (1.25–1.44) for 5–10 min, 1.39 (1.26–1.54) for 10–15 min, 1.20 (0.99–1.45) for 20–25 min, and 1.18 (0.80–1.73). The result for 25–30 min was 0.63 (0.29–1.38) and 0.44 (0.11–1.69) after 30 min, indicating that prehospital AAM within 15 min improves survival, similar to the results reported by Izawa et al. [28]. For neurological outcomes at 1 month, AAM did not contribute to outcome improvement regardless of the initial waveform. These studies suggest that prehospital AAM is effective only in non-shockable CA. One reason may be that respiratory failure and asphyxia are common causes of arrest in these conditions, although these findings need to be validated to further elucidate the underlying mechanism.

### 1.2. Adult vs. Pediatric Patients: Epidemiology, Prognosis, and Treatment Response

Several epidemiological differences between adult and pediatric patients who have experienced OHCA have been observed in relation to incidence, CA cause, and clinical course. Regarding incidence, adult CA occurs in 60–100 per 100,000 persons per year [1,30,31], whereas pediatric CA reports occur in 5–10 per 100,000 persons per year [32,33,34]. Regarding the cause of CA, shockable waveforms are generally observed in 15–30% of adult CA cases, with coronary artery disease being the most common cause [1,2,3,4]. In contrast, respiratory failure is the most common cause of pediatric CA. Furthermore, trauma and drowning are more common causes in pediatric CA, whereas shockable CA is less common, accounting for only 5–10% of cases [32,33,34].

Survival rates at discharge for adult OHCA cases have been reported to range from 13% to 15% in recent years, although there is variation across reports [35,36,37]. Conversely, survival rates at discharge for pediatric OHCA cases are generally lower, ranging from 5–9%, with some reports showing survival rates > 10% [38,39,40,41,42].

Neurologic outcomes at discharge for adult CA cases have been reported as favorable (cerebral performance category 1–2 or modified Rankin scale ≤ 3) in 50–95% of survivors. Furthermore, 80–90% of survivors experience excellent outcomes in developed countries and regions with well-established cardiopulmonary resuscitation systems, according to the 2020 World Resuscitation Congress [30]. For pediatric CA, the number of patients with favorable neurological outcomes at discharge (e.g., pediatric cerebral performance category 1–2) is lower than that of adults, ranging no higher than 10% [32,33,34,43,44]. Thus, pediatric patients who experience OHCA have higher mortality rates and poorer neurologic prognoses than adult patients. Several factors account for the disparity between pediatric and adult CA outcomes. First, trauma and drowning, which are common causes of pediatric CA, lead to poorer recovery chances. Second, children have less developed physiological compensatory mechanisms for hypoxia and shock compared with adults. Lastly, medical personnel may have less experience in managing critically ill pediatric patients, especially those in arrest.

Differences in treatment efficacy during resuscitation have also been reported between pediatric and adult CA cases. Prehospital AAM, while beneficial for adult non-shockable CA due to a higher chance of achieving ROSC and decreased mortality [26,28], has not shown the same benefits for pediatric patients with CA. To date, no high-quality RCTs have been undertaken in relation to pediatric patients; however, Amagasa et al. conducted a network meta-analysis on AAM in 2023 [45]. This network meta-analysis compared bag-mask ventilation [BMV] with SGA and ET intubation in 4852 patients across five studies (one intervention and four observational studies). For survival at discharge or at 1 month, the application of BMV was associated with higher survival rates compared with ET intubation (RR 0.44, 95% CI 0.25–0.77). No differences were found for other comparisons. There were also no significant differences in favorable neurological outcomes at discharge or at 1 month for any of the interventions. They concluded that the current evidence favors BMV for pediatric AAM. For adrenaline, recent high-quality studies have reported improved survival but that adrenaline administration is not prognostic for neurological outcomes in adult CA [25,26]. As with AAM, there are no RCTs on adrenaline regarding pediatric CA; however, Oshimo et al. conducted a meta-analysis based on seven observational studies in 2021 [46], which evaluated the timing of adrenaline administration and compared the time to the first dose of epinephrine in pediatric OHCA: <15 min vs. >15 min. For survival at discharge, a time to epinephrine administration of <15 min was significantly associated with a favorable outcome (RR 2.49, 95% CI 1.30–4.77). For favorable neurologic outcomes, a time to epinephrine administration of <15 min tended to improve outcomes, although the difference was not significant (RR 3.94, 95% CI 0.99–15.64).

These studies, therefore, suggest that resuscitation strategies must be tailored to the individual needs of adult and pediatric OHCA cases based on the underlying causes of CA. Moreover, this review also indicates that there are few high-quality studies, including RCTs, on resuscitative treatment for pediatric OHCA and that further investigation is needed.

### 1.3. Differential Severity of PCAS: Effects of TTM

Debates regarding the superiority of hypothermia or normothermia in TTM for patients with post-CA syndrome (PCAS) remain inconclusive. Recently, two high-quality RCTs reported contrasting findings. In 2019, the HYPERION trial, a multicenter study involving 25 French intensive care units that focused on non-shockable CA, reported a significantly higher percentage of patients who survived with good neurological outcomes at 90 days in a hypothermia group (33 °C) compared to a normothermia group (37 °C) [47]. In contrast, the TTM-2 OHCA trial, comprising 70% with shockable CA and 30% with non-shockable CA, reported no differences in mortality or neurological outcomes at 6 months between hypothermia (33 °C) and normothermia (<37.8 °C) groups [48]. This difference could be explained as owing to variations in disease severity among the enrolled patients, given the differences in certain baseline characteristics, such as the percentage of patients in shock on admission (29% vs. 58%, respectively). Further studies are required to identify which types of disease severity benefit more from therapeutic hypothermia [49].

Several studies have also been conducted to evaluate the effectiveness of therapeutic hypothermia based on PCAS severity (Table 2). In 2020, Callaway et al. conducted a study where patients with PCAS were classified into four severity levels (1–4), according to the Pittsburgh cardiac arrest category (PCAC). Patient outcomes following hypothermia and normothermia treatment were then compared in terms of severity [50]. Patients with the most severe PCAS (PCAC 4) showed higher survival rates with hypothermia, whereas those with mild-to-moderate PCAS (PCAC 2–3) showed higher survival rates with normothermia.

Another study evaluating the effect of therapeutic hypothermia based on disease severity made use of the r-CAST score, a risk assessment tool for PCAS [51]. Patients were classified as mild, moderate, or severe, and the effects of hypothermia (33–34 °C) and normothermia (35–36 °C) were compared for each severity level. Notably, no differences in neurological outcomes or mortality were observed between hypothermia and normothermia in patients with mild or severe PCAS. However, therapeutic hypothermia resulted in lower mortality rates and better neurological outcomes at 30 days among patients with moderate PCAS.

In 2022, Nutma et al. utilized electroencephalography (EEG) to estimate the varied effects of therapeutic hypothermia according to PCAS severity [52]. Specifically, they classified patients into three levels based on EEG patterns (mild, moderate, and severe) and compared the effects of hypothermia (33 °C) and normothermia (36 °C) for each severity level. Similar to the r-CAST study, the prognosis was similar between patients with mild and severe PCAS and with hypothermia and normothermia, whereas patients with moderate PCAS with hypothermia showed better neurological outcomes.

Furthermore, Lasccarou et al. compared the same parameters in patients who were categorized into three stages according to a modified CA hospital prognosis classification for PCAS and showed that the effect of TTM (32–36 °C) depended on disease severity [53]. An RCT is currently underway to investigate the effect of therapeutic hypothermia for a particular severity group, the results of which are anticipated to further enhance understanding of this matter [54].

This study had some limitations. It did not fully cover all literature related to this research field. While we considered the heterogeneity of OHCA in terms of three important factors, we did not explore other factors such as regional differences. Furthermore, other confounding factors may have been involved in OHCA, which we did not discuss. For example, differences in the availability of emergency medical services could contribute to differences in patient outcomes. Despite these limitations, we were able to show differences in treatment effects based on the heterogeneity of patients with OHCA and suggest options for better tailored treatment for patients. We consider this study to be likely to assist in guiding future OHCA treatment and management.

## 2. Conclusions

We reviewed literature on the heterogeneity of OHCA from different perspectives. Previous studies underscore the possibility that the clinical course and treatment effects may vary depending on the initial CA waveform, age, and PCAS severity. Further clinical and pathological research is essential to improve the prognosis for patients with OHCA.

## Figures and Tables

**Table 2:** A study comparing the effects of Targeted temperature management in different severities of PCAS.

Study Year	Design	Comparison	Severity	Results
Callaway et al., 2020 [50]	Observational study	Hypothermia vs. Normothermia	PCAC score	Severe: Survival was higher in the hypothermia. Mild and Moderate: No difference between two groups
Nishikimi et al., 2021 [51]	Observational study	Hypothermia vs. Normothermia	r-CAST score	Moderate: Neurological outcome was better in hypothermia. Mild and Severe: No difference between two groups
Nutma et al., 2022 [52]	Observational study	Hypothermia vs. Normothermia	EEG	Moderate: Neurological outcome was better in the hypothermia. Mild and Severe: No differences between two groups.
Lasccarou et al., 2023 [53]	Observational study	Hypothermia vs. Normothermia	mCAHP score	Mild and Severe: Neurological outcome was better in the TTM group. Moderate: No differences between two groups

TTM, targeted temperature management; PCAS, post-cardiopulmonary arrest syndrome; PCAC, Pittsburgh Cardiac Arrest Category; r-CAST, revised-cardiac arrest syndrome for induced therapeutic hypothermia; EEG, electroencephalogram; mCAHP, the modified version of the Cardiac Arrest Hospital Prognosis.

## Data Availability

The original contributions presented in the study are included in the article, further inquiries can be directed to the corresponding author/s. The raw data supporting the conclusions of this article will be made available by the authors on request.
